# Detection of monoclonal immunoglobulin heavy chain gene rearrangement (FR3) in Thai malignant lymphoma by High Resolution Melting curve analysis

**DOI:** 10.1186/1746-1596-5-31

**Published:** 2010-05-19

**Authors:** Tanawan Kummalue, Anchalee Chuphrom, Sanya Sukpanichanant, Tawatchai Pongpruttipan, Sathien Sukpanichanant

**Affiliations:** 1 Department of Clinical Pathology, Faculty of Medicine Siriraj Hospital, Bangkok 10700, Thailand; 2 Department of Pathology, Faculty of Medicine Siriraj Hospital, Bangkok 10700, Thailand

## Abstract

**Abstract:**

Malignant lymphoma, especially non-Hodgkin lymphoma, is one of the most common hematologic malignancies in Thailand. The diagnosis of malignant lymphoma is often problematic, especially in early stages of the disease. Detection of antigen receptor gene rearrangement including T cell receptor (TCR) and immunoglobulin heavy chain (IgH) by polymerase chain reaction followed by heteroduplex has currently become standard whereas fluorescent fragment analysis (GeneScan) has been used for confirmation test. In this study, three techniques had been compared: thermocycler polymerase chain reaction (PCR) followed by heteroduplex and polyacrylamide gel electrophoresis, GeneScan analysis, and real time PCR with High Resolution Melting curve analysis (HRM). The comparison was carried out with DNA extracted from paraffin embedded tissues diagnosed as B- cell non-Hodgkin lymphoma. Specific PCR primers sequences for IgH gene variable region 3, including fluorescence labeled IgH primers were used and results were compared with HRM. In conclusion, the detection IgH gene rearrangement by HRM in the LightCycler System showed potential for distinguishing monoclonality from polyclonality in B-cell non-Hodgkin lymphoma.

**Introduction:**

Malignant lymphoma, especially non-Hodgkin lymphoma, is one of the most common hematologic malignancies in Thailand. The incidence rate as reported by Ministry of Public Health is 3.1 per 100,000 population in female whereas the rate in male is 4.5 per 100,000 population [1]. At Siriraj Hospital, the new cases diagnosed as malignant lymphoma were 214.6 cases/year [2]. The diagnosis of malignant lymphoma is often problematic, especially in early stages of the disease. Therefore, detection of antigen receptor gene rearrangement including T cell receptor (TCR) and immunoglobulin heavy chain (IgH) by polymerase chain reaction (PCR) assay has recently become a standard laboratory test for discrimination of reactive from malignant clonal lymphoproliferation [3,4]. Analyzing DNA extracted from formalin-fixed, paraffin-embedded tissues by multiplex PCR techniques is more rapid, accurate and highly sensitive. Measuring the size of the amplicon from PCR analysis could be used to diagnose malignant lymphoma with monoclonal pattern showing specific and distinct bands detected on acrylamide gel electrophoresis. However, this technique has some limitations and some patients might require a further confirmation test such as GeneScan or fragment analysis [5,6].

GeneScan technique or fragment analysis reflects size and peak of DNA by using capillary gel electrophoresis. This technique is highly sensitive and can detect 0.5-1% of clonal lymphoid cells. It measures the amplicons by using various fluorescently labeled primers at forward or reverse sides and a specific size standard. Using a Genetic Analyzer machine and GeneMapper software (Applied Bioscience, USA), the monoclonal pattern revealed one single, sharp and high peak at the specific size corresponding to acrylamide gel pattern, whereas the polyclonal pattern showed multiple and small peak condensed at the same size standard. This technique is the most sensitive and accurate technique; however, it usually requires high technical experience and is also of high cost [7]. Therefore, rapid and more cost effective technique are being sought.

LightCycler PCR performs the diagnostic detection of amplicon via melting curve analysis within 2 hours with the use of a specific dye [8,9]. This dye consists of two types: one known as SYBR-Green I which is non specific and the other named as High Resolution Melting analysis (HRM) which is highly sensitive, more accurate and stable. Several reports demonstrated that this new instrument combined with DNA intercalating dyes can be used to discriminate sequence changes in PCR amplicon without manual handling of PCR product [10,11]. Therefore, current investigations using melting curve analysis are being developed [12,13].

In this study, three different techniques were compared to evaluate the suitability of LightCycler PCR with HRM as the clonal diagnostic tool for IgH gene rearrangement in B-cell non-Hogdkin lymphoma, i.e. thermocycler PCR followed by heteroduplex analysis and PAGE, GeneScan analysis and LightCycler PCR with HRM.

## Materials and methods

### Patients

Twenty-six cases of B-cell non-Hodgkin lymphoma diagnosed by hematopathologists from Department of Pathology, Faculty of Medicine Siriraj Hospital, Mahidol University, were enrolled in this study. The diagnosis of B-cell non-Hodgkin lymphoma followed the WHO classification 2008 by using morphological and immunophenotypical features. This study was approved by the Siriraj Institutional Review Board, Faculty of Medicine Siriraj Hospital, Mahidol University.

### DNA extraction

Serial 10 μm thick sections were obtained with a standard microtome and disposable blades from paraffin embedded lymph nodes. In brief, waxes were extracted with xylene followed by centrifugation at 6000 g for 10 minutes. After this initial step, pellets were resuspended in absolute ethanol for 5 minutes and centrifuged. Paraffin-free tissue was dried with heating box for 15 minutes at 37°C and subjected to the DNA extraction procedures using QIAamp DNA mini kit (Qiagen). The concentration of DNA was measured with Smartspect (BIO-RAD, USA).

### PCR primers and thermalcycler program

PCR primers sequences for IgH variable gene framework region 3 (FR3) plus joining region (JH) consensus primers were designed followed BIOMED-2 [14]. These primers were designed to target conserved DNA sequences surrounding the unique hypervariable anigen-binding complementarity determining region 3 (CDR3). To amplify the IgH variable region in Thermalcycler (BIO-RAD, USA), genomic DNA at 800-1000 ng. was added up to a final reaction 50 μl. After the initial "hot start" using Faststart (Roche Diagnostic) at 95°C for 7 minutes, PCR cycle program included: denaturing at 95°C for 45 seconds, followed by annealing at 60°C for 45 seconds, and extension at 72°C for 90 seconds. The program was repeated for 34 cycles. A final extension was performed at 72°C for 10 minutes.

### Heteroduplex analysis

For heteroduplex analysis, the PCR products 20 μl were denatured at 95°C for 5 minutes, and then rapid random renaturation at 4°C for 1 hour. Heteroduplex were detected by polyacrylamide gel electrophoresis (PAGE) on an 6% non-denaturing acrylamide gel (16 × 16 × 0.1 cm) using SE600 Ruby (Amersham Bioscience, USA) at room temperature for 210 minutes at 110 Volts. The gel was then stained with 0.5 μg/ml ethidium bromide and photographed under ultraviolet light using ChemiDoc™XRS System (BIO-RAD, USA). PCR and heteroduplex analysis were performed in duplicate.

### LightCycler PCR and High Resolution Melting curve analysis (HRM)

The PCR product of IgH variable region 3 was further amplified in the LightCycler System 480 (Roche Diagnostic). The reaction mixture for each well contained high resolution melting master 10 μl, 5 pmol/μl of IgH (FR3) primer 2 μl, 25 mM of MgCl_2 _2.4 μl, dH_2_O PCR grade 4.6 μl, and diluted 1:100 PCR product 1 μl, was added up to a final volume of 20 μl. After the initial "hot start", PCR cycle parameters were held at pre-incubator 95°C for 10 minutes, and amplification program was 95°C 10 second, 55°C 15 seconds, and 72°C 15 seconds. High resolution melting program was 95°C 1 minute, 55°C 1 minute, 70°C 1 second. After the 30 PCR amplification cycles, the LightCycler System DNA melting curve was analysed by LightCycler 480 software. All samples were performed in duplicate.

### Fluorescent fragment analysis (GeneScan)

For GeneScan analysis, the PCR product was carried out using the conditions mentioned above with fluorescence labeled primers. The sequences of fluorescence labeled IgH primers were as follows:

HVF3-3FL 5'-TCTGCAAATGAACAGCCTGAGAGCC-3' Dye-label 5' VIC,

HVF4-3FL 5'-GAGCTCTGTGACCGCCGCGGACACG-3' Dye-label 5' VIC,

HVF5-3FL 5'-CAGCACCGCCTACCTGCAGTGGAGC-3' Dye-label 5' NED,

HVF6-3FL 5'-GTTCTCCCTGCAGCTGAACTCTGTG-3' Dye-label 5' NED,

HVF7-3FL 5'-CAGCACGGCATATCTGCAGATCAG-3' Dye-label 5' PET,

HDF1-3FL 5'-TGGAGCTGAGCAGCCTGAGATCTGA-3' Dye-label 5' 6FAM, and HVF2-3FL 5'-CAATGACCAACATGGACCCTGTGGA-3' Dye-label 5' 6FAM. The amplified fragments were analyzed by ABI 3130 Genetic Analyzer (Applied Bioscience, USA) according to the manufacturer's protocol. In brief, each sample contained 1 μl of the PCR product mixed with 10.7 μl of Hi-Di formamide and 0.3 μl of 600 LIZ^® ^size standard (Applied bioscience, USA). Samples were denatured at 95°C for 5 minutes and immediately chilled on ice for 2 minutes. The gel used for capillary electrophoresis was POP-4 polymer solution (Applied Bioscience, USA). Results were visualized using the ABI GeneMapper v 3.2 software (Applied Bioscience, USA). The expected size varied from 100-170 bp.

## Results

### Patients characteristics

Age of the patients in this study ranged from 13 to 79 years. As shown in Table 1, of 26 patients enrolled in this study, 2 patients were diagnosed as Burkitt lymphoma (BL), 4 cases were extranodal marginal zone lymphoma of mucosa-associated lymphoid tissue (MALT) lymphoma, 3 cases were follicular lymphoma (FL), 2 cases were nodal marginal zone lymphoma, and the other 15 cases were diffuse large B cell lymphoma (DLBCL). From previous report, the 5 most common types of B-cell non Hodgkin lymphoma in Thailand are DLBCL, FL, MALT lymphoma, BL, and small lymphocytic lymphoma (SLL)[2]. Therefore, this finding is in accordance with the distribution that was observed before. Percentage of lymphoma involvement within each specimen was demonstrated in the Table 1.

**Table 1:** Summary of cases analyzed by PAGE, GensScan and LightCycler.

Patient	Age/Sex	Tissues	Diagnosis	GeneScan	HRM	% tumor/B cell*
1	58/F	Thyroid gland	Diffuse large B cell	Monoclonal H = 8818	Monoclonal Tm = 84.83	90

2	46/M	Lymph node	Diffuse large B cell	Monoclonal H = 8575	Monoclonal Tm = 85.18	95

3	51/F	Soft tissue, submandibular	Diffuse large B cell	Monoclonal H = 8814	Polyclonal Tm = 83.42/86.85	95

4	77/F	Lymph node	Diffuse large B cell	Monoclonal H = 7831	Monoclonal Tm = 88.33	90

5	49/M	Stomach	Diffuse large B cell	Monoclonal H = 9008	Monoclonal Tm = 86.04	90

6	63/F	Lymph node	Diffuse large B cell	Monoclonal H = 7759	Polyclonal Tm = 83.26/88.62	75

7	68/M	Lymph node	Diffuse large B cell	Monoclonal H = 8405	Monoclonal Tm = 85.59	95

8	42/M	Lymph node	Diffuse large B cell	Monoclonal H = 6731	Monoclonal Tm = 86.06	90

9	68/F	Skin	Diffuse large B cell	Monoclonal H = 1599	Monoclonal Tm = 85.01	85

10	58/M	Skin	Diffuse large B cell	Monoclonal H = 8060	Monoclonal Tm = 86.36	95

11	68/F	Lymph node	Diffuse large B cell	Monoclonal H = 8005	Monoclonal Tm = 87.24	90

12	35/F	Tonsil	Follicular lymphoma	Monoclonal H = 8738	Monoclonal Tm = 88.85	90

13	70/F	Lacrimal gland	Extranodal marginal zone B cell	Monoclonal H = 8122	Monoclonal Tm = 80.62	95

14	61/M	Lymph node	Follicular lymphoma	Monoclonal H = 6887	Polyclonal Tm = 84.00/87.01	75

15	67/F	Lymph node	Marginal zone B cell	Monoclonal H = 7862	Monoclonal Tm = 86.10	80

16	72/M	Tonsil	Extranodal marginal zone B cell	Monoclonal H = 7004	Monoclonal Tm = 86.15	95

17	13/M	omentum	Burkitt lymphoma	Monoclonal H = 7936	Monoclonal TM = 86.48	99

18	67/F	Soft tissue, cheek	Extranodal marginal zone B cell	Monoclonal H = 8186	Monoclonal Tm = 86.34	95

19	60/F	Lymph node	Diffuse large B cell	Monoclonal H = 7805	Monoclonal Tm = 86.8	99

20	79/F	Lymph node	Marginal zone B cell lymphoma	Monoclonal H = 9120	Monoclonal Tm = 87.29	90

21	75/M	Thyroid gland	Extranodal marginal zone B cell	Monoclonal H = 8418	Monoclonal Tm = 87.37	90

22	73/F	Soft tissue, neck	Diffuse large B cell	Monoclonal H = 7921	Monoclonal Tm = 85.59	95

23	55/M	Lymph node	Burkitt lymphoma	Monoclonal H = 8423	Monoclonal Tm = 86.03	99

24	72/F	Nasal cavity	Diffuse large B cell	Monoclonal H = 8974	Monoclonal Tm = 85.55	98

25	N/M	Lymph node	Diffuse large B cell	Monoclonal H = 7742	Monoclonal Tm = 88.44	95

26	63/F	Lymph node	Follicular lymphoma	Monoclonal H = 8977	Monoclonal Tm = 87.14	90

H = Height of peak in GeneScan, N = no record

(* % tumor/B cell means the percentage of tumor cells when the total B cell population are concerned.)

### High Resolution Melting curve analysis and lower limit for detecting monoclonal IgH gene rearrangement

The optimal number of PCR cycles was evaluated using monoclonal sample and polyclonal sample from positive lymph node and tonsil, respectively. Based on previous report, 30 cycles of LightCycler PCR showed the Tm with peak height at least twice as high, and less than one half as wide as a polyclonal tonsil sample. Therefore, the number of PCR at 30 cycles was used to investigate in this study [15].

**Figure 1:**
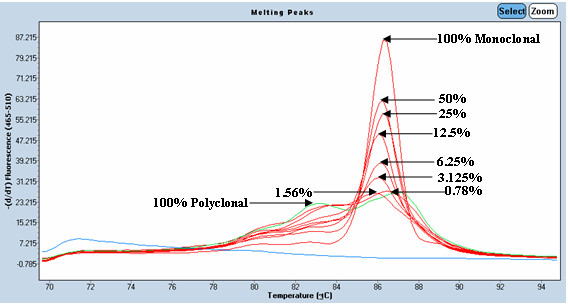
**Detection limit of LightCycler System using melting curve analysis**. DNA of positive sample was diluted with tonsil DNA into 50%, 25%, 12.5%, 6.25%, 3.125%, 1.56%, and 0.78%. At the percentage equal to 0.78, Tm was still detected as monoclone.

**Figure 2:**
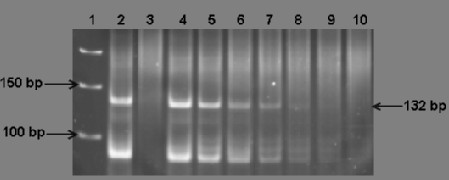
**Minimal detection of polyacrylamide gel electrophoresis analysis and ethidium bromide staining of monoclonal IgH gene rearrangement**. The distinct band consistent with monoclonal IgH gene rearrangement could be detected at concentration as low as 3.125%. Lane 1 = GeneRuler 50 bp DNA ladder marker, Lane 2 = positive control, Lane 3 = negative control, Lane 4-10 = serial dilutions 50%, 25%, 12.5%, 6.25%, 3.125%, 1.56%, and 0.78%, respectively.

**Figure 3:**
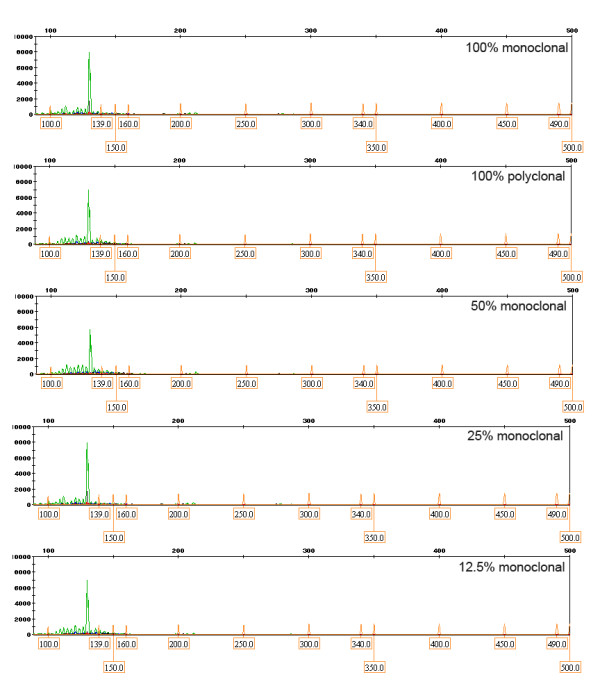
**Detection of minimal dilution for IgH monoclonality by GeneScan**. The one prominent peak could be detected at concentration as low as 0.78%.

**Figure 4:**
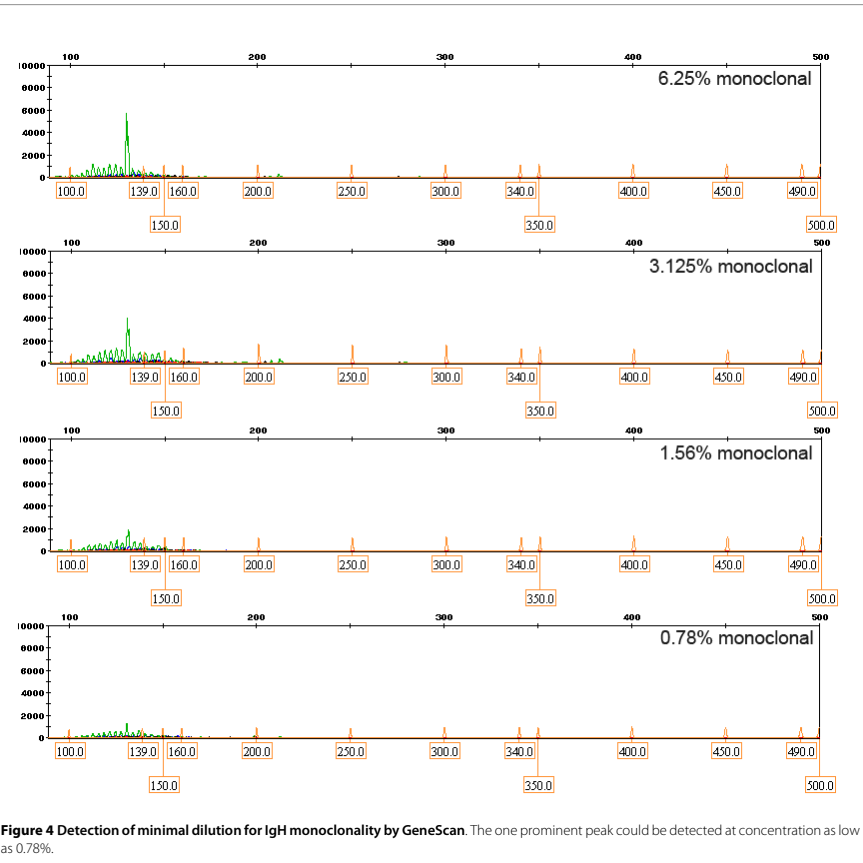
**Detection of minimal dilution for IgH monoclonality by GeneScan**. The one prominent peak could be detected at concentration as low as 0.78%.

To evaluate the minimum detectable percentage of IgH monoclonal DNA with melting curve analysis, serial dilutions of IgH tube C monoclone with polyclone at various percentages of 50%, 25%, 12.5%, 6.25%, 3.125%, 1.56%, and 0.78% were performed. After 30 cycles in LightCycler, Tm at a percentage equal 0.78% was still detected as monoclonal group by melting curve analysis as shown in Figure 1. For PAGE, one discrete band at specific size could be detected at concentration 3.125% whereas a distinct peak in GeneScan was clearly demonstrated at concentration as low as 0.78% as shown in Figure 2, Figure 3 and Figure 4.

### Comparison of PAGE and GeneScan

**Figure 5:**
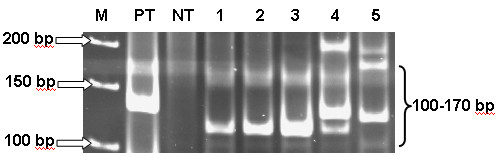
**IgH gene rearrangement showing the distinct band at 100-170 bp on ethidium bromide stained acrylamide gel**. (arrow) M = GeneRuler 50 bp DNA Ladder Marker (Fermentas), PT = Positive control, NT = Negative control, 1-5 = Patients samples.

**Figure 6:**
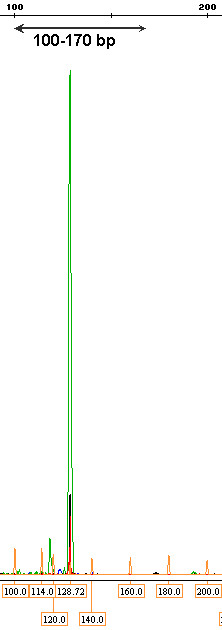
**GeneScan analysis of IgH monoclonal sample revealed 1 dominant peak in the profile showing the monoclonal pattern**.

All 26 IgH monoclonal samples showed a sharp band on ethidium bromide stained acrylamide gel with 100-170 bp as demonstrated in Figure 5. In GeneScan, all DNA monoclonal samples showed products of IgH which were characterized by 1 or 2 dominant peaks in the GeneScan profile, indicating the presence of B cell clonal proliferation as shown in Figure 6. As a result, all 26 samples in this study were confirmed to be monoclonality by GeneScan analysis.

### Comparison of GeneScan and High Resolution Melting curve analysis

**Figure 7:**
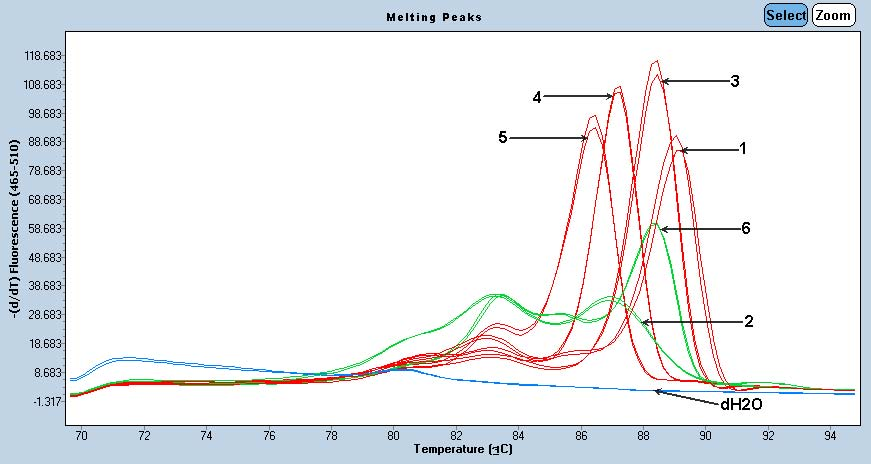
**Demonstrated melting curve analysis in IgH malignant lymphoma using LightCycler System**. (arrow) 1 = positive monoclone, 2 = negative polyclone, 3 = patient sample (monoclone), 4 = patient sample (monoclone), 5 = patient sample (monoclone), 6 = patient sample (polyclone).

Comparing GeneScan versus High Resolution Melting curve analysis was performed. In the melting curve analysis by HRM, 23 samples demonstrated a high and narrow DNA melting curve that was greater twice the peak height but less than one half the width, when compared with the polyclonal sample as shown in Figure 7. Notably, 3 samples showed polyclonal pattern in melting curve analysis.

## Discussion

Immunoglobulin heavy chain gene rearrangement has been widely used for diagnosis of B cell monoclonality [16]. At present, detection of monoclonal B cell population is based on heteroduplex including fluorescent PCR with GeneScan analysis. Recently, melting curve analysis (Tm) of duplex DNA has been applied to distinquish the monoclonal from polyclonal population. In this study, three techniques: thermocycler PCR followed by heteroduplex and PAGE, GeneScan analysis and LightCycler PCR with HRM, were investigated in terms of detecting monoclonal immunoglobulin heavy chain variable gene framework region 3 (FR3).

From melting temperature of duplex DNA, 3 samples accounting for 11.5% revealed false negative by expressing polyclonality when compared with GeneScan. No false positive was detected in our series. From a previous report, using PCR-MCA (LightCycler Technology with high-speed amplification, and Idaho-Technology with rapid and high-resolution melting curve analysis -MCA) showed high sensitivity and specificity at 89.2% and 100% respectively [17]. Another study of 10 gastric MALT lymphomas using melting curve analysis with SYBR Green I showed similar sensitivity and specificity at 95.2% and 100%, respectively [18]. This investigation also demonstrated high sensitivity as compared to other reports. The detection of the minimum percentage of monoclonality by HRM in our series was at 0.78% dilution which was higher than previous report [18]. 88.5% sensitivity and 100% specificity were revealed in HRM indicating that a positive result in this method is specific for IgH monoclonality, whereas sensitivity and specificity of PAGE and GeneScan were considered as 100%.

LightCycler technology has been used in many areas of clinical testing because of its rapid turn around time and accuracy. High Resolution Melting curve analysis, which is faster, simpler and less expensive, was developed as the latest method for product analysis in molecular diagnostics [19,20]. As compared all three methods concerning quality and accuracy, GeneScan with specific fluorescence labeled primers and HRM could detect the minimum level as low as 0.78%. However, GeneScan has the highest cost which is approximately 3 times higher when compared with PAGE, and about 2 times when compared with HRM. In conclusion, based on our study, High Resolution Melting curve analysis in LightCycler System still has a potential role for clinically demonstrating monoclonal IgH.

## Competing interests

The authors declare that they have no competing interests.

## Authors' contributions

TK designed the experiments, controlled the overall project, and wrote the manuscript. AC carried out the experiments. SS^2 ^and TP participated in sending the tissue blocks. SS^1 ^supported some instruments. All authors read and approved the final manuscript.
